# Long-Term Outcomes of Cultivated Limbal Epithelial Transplantation: Evaluation and Comparison of Results in Children and Adults

**DOI:** 10.1155/2015/480983

**Published:** 2015-12-03

**Authors:** Anita Ganger, M. Vanathi, Sujata Mohanty, Radhika Tandon

**Affiliations:** ^1^Cornea, Refractive and Ocular Surface Rehabilitation Services, Dr. Rajendra Prasad Centre for Ophthalmic Sciences, All India Institute of Medical Sciences, New Delhi 110029, India; ^2^Centre of Excellence for Stem Cell Research, Dr. Rajendra Prasad Centre for Ophthalmic Sciences, All India Institute of Medical Sciences, New Delhi 110029, India

## Abstract

*Purpose.* To compare the long-term clinical outcomes of cultivated limbal epithelial transplantation (CLET) in children and adults with limbal stem cell deficiency.* Design*. Retrospective case series.* Methods*. Case records of patients with limbal stem cell deficiency (LSCD) who underwent CLET from April 2004 to December 2014 were studied. Outcome measures were compared in terms of anatomical success and visual improvement. Parameters for total anatomical success were avascular, epithelized, and clinically stable corneal surface without conjunctivalization, whereas partial anatomical success was considered when mild vascularization (sparing centre of cornea) and mild conjunctivalization were noted along with complete epithelization.* Results*. A total of 62 cases underwent the CLET procedure: 38 (61.3%) were children and 24 (38.7%) were adults. Patients with unilateral LSCD (33 children and 21 adults) had autografts and those with bilateral LSCD (5 children and 3 adults) had allografts. Amongst the 54 autografts partial and total anatomical success were noted in 21.2% and 66.6% children, respectively, and 19.0% and 80.9% in adults, respectively (*p* value 0.23). Visual improvement of 1 line and ≥2 lines was seen in 57.5% and 21.2% children, respectively, and 38% and 38% in adults, respectively (*p* value 0.31).* Conclusion*. Cultivated limbal epithelial transplantation gives good long-term results in patients with LSCD and the outcomes are comparable in children and adults.

## 1. Introduction

The cornea is the most important part of ocular surface which provides eye with two-thirds of its focusing power and protection of ocular integrity and helps in maintaining clear and useful vision due to its transparent and avascular nature [1]. Corneal stem cells play important anti-inflammatory as well as antiangiogenic roles in maintaining a normal corneal microenvironment and destruction of which leads to limbal stem cell deficiency [2]. Two major associations of primary limbal stem cell deficiency are aniridia and ectodermal dysplasia. For secondary or acquired limbal stem cells deficiency physical, chemical, thermal, and immunological insults are the major factors out of which chemical injuries contribute the maximum [3].

A number of therapeutic strategies for the treatment of LSCD have been documented in the past, but the decision for choosing a particular treatment modality depends solely on the severity and laterality of deficiency [4, 5]. In limbal stem cell deficiency as well as in other ocular pathologies, the ocular inflammatory response to any kind of insult is always expected to be more in the paediatric age group [6]. In most of the published literature the success rates of CLET have been documented in adult patients, except one study where the surgical outcomes of CLET were reported in paediatric age group only [7]. The present study was hence undertaken to compare the long-term results of cultivated limbal epithelial transplant in children versus adult patients, which has not been documented yet.

## 2. Materials and Methodology

The study was conducted in accordance with the tenets of the Declaration of Helsinki, with permission from the Institutional Ethics Committee, AIIMS. Retrospectively follow-up data of consecutive LSCD patients with either partial or total limbal stem cell deficiency who underwent CLET from April 2004 to December 2014 was reviewed. To compare the outcome results of CLET in children and adults, patients were divided into two groups: <15 years old patients were considered as children and patients of ≥15 years of age were included in adult group. The surgical outcome results were compared in children as well as adults in terms of anatomical success and visual improvement.

Two surgeons (Professor Radhika Tandon and Dr. M. Vanathi) had performed all surgeries by following a standardized protocol.

### 2.1. Retrospective Surgical Procedure Details and Follow-Up Protocol Were Checked from Hospital Records

#### 2.1.1. Method Followed for Preparation of Human Amniotic Membrane

Amniotic membranes were obtained after caesarian section delivery, after ensuring sterile conditions and seronegativity of donor (HIV, hepatitis B surface antigen, hepatitis C virus, and syphilis). The amnion and chorion were separated from the placenta and placed in a sterile container containing normal saline (540 mL) with dexamethasone (80 mg) and heparin (5000 IU). Placenta was washed to remove blood clots with balanced saline solution containing 50 micrograms/mL of streptomycin and 2.5 micrograms/mL of amphotericin B under a laminar flow hood. Followed by washing, separation of amniotic membrane from chorion was done by blunt dissection. The amniotic membrane was again thoroughly washed to remove all residual blood and debris and then placed on a sterile flat tray with the stromal side up and nitrocellulose paper discs were placed over it following which the membrane was cut around the paper discs to prepare 4 × 4 cm circular pieces and the edges wrapped around the margin of the paper and stored in Dulbecco's modified Eagle's medium along with glycerol AR grade, at −80°C in a deep freezer.

#### 2.1.2. Technique Followed for Autograft and Allograft Transplantation

Surgery for autograft transplantation included first-stage limbal stem cell lenticule (2 × 2 mm) extraction from contralateral eye in case of unilateral LSCD. Second-stage surgery was started with the dissection of the ocular surface pannus 2 to 3 mm behind the limbus with the help of conjunctival scissors or number 15 blade on a Bard-Parker handle and sent for histopathological examination. Cautery was done to achieve adequate haemostasis after symblepharon release. The human amniotic membrane having cultivated limbal epithelium cells over it was gently unfolded over the cornea as well as limbus with epithelial side up. The membrane was secured to the ocular surface with either 10-0 monofilament nylon material sutures or fibrin glue. A bandage contact lens was applied at the end.

#### 2.1.3. For Allograft

All cadaveric eyes were received either as a whole globe in a moist chamber or as a corneoscleral ring in corneal preservation medium from the Eye Bank. They were harvested and procured with at least a 5 mm, 360-degree conjunctival mantle. Cadaveric eyes were used within first or second postmortem day after getting negative serological screening report for infectious diseases. With Westcott scissors and a number 15 sterile surgical blade all harvested corneoscleral rings obtained after corneal grafts were trephined from the endothelial side by using 7 to 8 mm disposable corneal trephines which were dissected to obtain limbal lenticules for* ex vivo* expansion. In the recipient eye the procedure followed was similar to autograft transplantation. After allograft transplantation oral steroids 1 mg/kg body weight had been given and gradually tapered over 3 months. Along with oral steroids, 1% topical cyclosporine (QID) had been given for six months. Systemic immunosuppressive agents had not been given due to affordability issues.

Among all the included patients autografts were performed in majority of the patients, with unilateral LSCD, whereas allografts were done in few patients only, who had bilateral total LSCD.

### 2.2. Outcome Measures

The outcome measures were compared in children as well as adults in terms of anatomical success and visual improvement. The outcome parameters for total anatomical success were avascular, epithelized, and clinically stable corneal surface without conjunctivalization, whereas partial anatomical success was considered when mild vascularization (not reaching up to the centre of the cornea) and mild conjunctivalization were noted along with complete epithelization.

Improvement in best corrected visual acuity (BCVA) was documented, in terms of either 1 line or ≥2 lines improvement after transplantation. The improvement in visual acuity was noted as visual success.

Failure of surgery was defined as absence of surface epithelization even after six weeks of surgery with progressive corneal conjunctivalization/vascularization.

The extent of limbal stem cell deficiency was considered while evaluating anatomical and visual success. Patients with limbal stem cell deficiency of ≤9 clock hours were included in partial LSCD group whereas patients with >9 clock hours involvement were included in total LSCD group.

Based on the interval between injury to limbal stem cells and surgery performed, patients were divided into 2 groups. Group 1 included patients operated ≤6 months of duration, whereas in group 2 this duration was >6 months.

Final outcomes were noted at 1 year postoperatively, though patients continued to remain under follow-up to evaluate long-term effects and capture any late complications.

### 2.3. Data Collection

Retrospectively, parameters like visual acuity, biomicroscopic examination findings, intraocular pressure values, and fundus details were noted from the records and files. The recorded findings at 1 week, 1 month, 3 months, 6 months, and 1 year were noted. Whole clinical data along with clinical photographs was analyzed from the records thoroughly.

### 2.4. Data Analysis

Study data was recorded in Excel Spreadsheet and statistical analysis was done by StataCorp LP 2013 (version 2013). Categorical variables were analyzed by chi square test. *p* value < 0.05 was taken as significant.

## 3. Results

Out of 110 patients' records, 62 long-term follow-up records were included in this analysis. The remaining 48 patients were excluded because of lack of sufficient follow-up data, though short-term data of these patients had been published already in 2011 [8]. Retrospective analysis of total 62 LSCD patients who underwent autograft/allograft limbal stem cell transplantation was done.

In 54 autograft patients, the mean age of the patients, mean follow-up period, and mean time duration between insult and the transplantation were 14.7 ± 10.0 years, 21.36 ± 17.80 months, and 9.91 ± 5.67 months, respectively, out of which 41 (75.9%) patients were males and 13 (24%) were females.

Amongst 54 autograft patients, 33 (61.1%) patients were <15 years (children) and 21 (38.9%) patients were ≥15 years of age (adults). Etiology of the LSCD in adult patients included thermochemical injury in 19 (90.5%) patients, operated ocular surface squamous neoplasia in 1 (4.8%) patient, and Stevens-Johnson syndrome in 1 (4.8%) patient, whereas in children thermochemical injury was the causative factor in all 33 children (Table 1).

**Figure 1:**
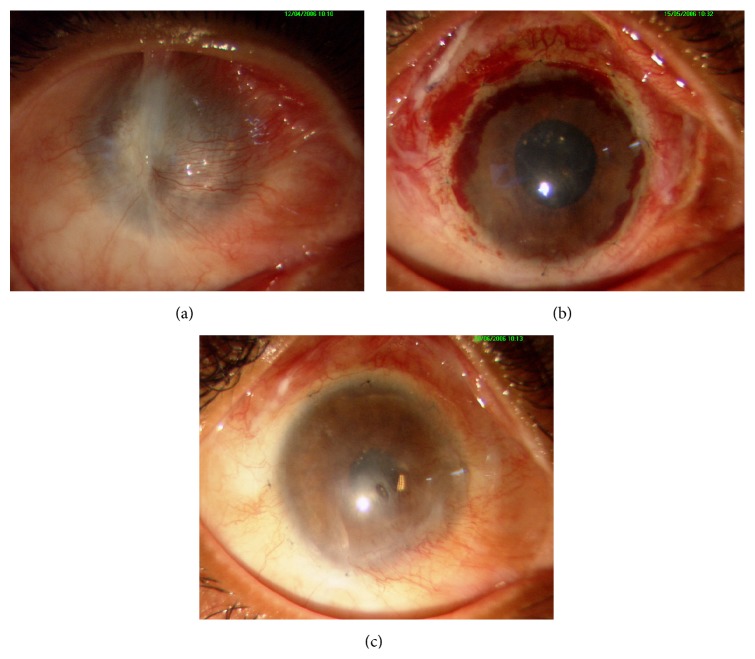
Representing a child (11-year-old male) who underwent autologous CLET for unilateral total LSCD, induced by chemical injury in right eye. (a) Preoperative clinical picture showing 360-degree conjunctivalization and vascularization. (b) One-month postoperative clinical picture showing improved ocular surface. (c) Clinical picture at 6 months.

**Figure 2:**
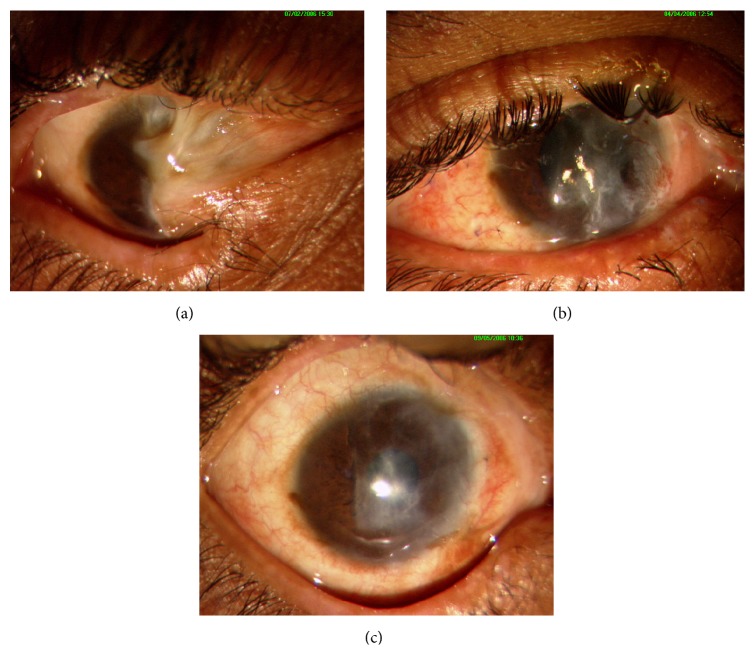
Representing adult patient (45-year-old male) who underwent autologous CLET for bilateral partial LSCD. (a) Preoperative clinical picture showing LSCD involving around 6 hours along with symblepharon inferonasally in RE. (b) Improved ocular surface with fornix formation at 3 months postoperatively. (c) Six-month postoperative clinical picture showing anatomical success.

In children anatomical success either partial or total was seen in 7 (21.2%) and 22 (66.6%), respectively, whereas failure was noted in 4 (12.1%) children. Visual success in terms of improvement of visual acuity of 1 line or ≥2 lines was noted in 19 (57.5%) and 7 (21.2%), whereas failure was seen in 7 (21.2%) patients. In adults anatomical success either partial or total was seen in 4 (19.0%) and 17 (80.9%), respectively, whereas failure was not noted in any of the adult patients. Visual success in terms of improvement of visual acuity of 1 line or ≥2 lines was noted in 8 (38.0%) and 8 (38.0%), respectively, after transplantation, whereas failure was seen in 4 (19.0%) patients. Comparison of anatomical success, visual success, and failure rates between children and adults was analyzed by chi square test as shown in Table 2. No statistically significant difference was found between children and adults, *p* values 0.23 and 0.31 for anatomical and visual success, respectively. Preoperative and postoperative clinical pictures of both paediatric and adult patient are shown in Figures 1 and 2, respectively.

Among 8 allograft patients, mean age, mean follow-up period, and mean time duration between insult and the transplantation were 15.88 ± 10.439 years, 24.7 ± 14.569 months, and 8.75 ± 4.268 months, respectively. Anatomical success either partial or total was seen in 12.5% and 50%, respectively, whereas failure was noted in 37.5% of cases. Visual success of more than 1-line improvement or ≥2-line improvement was seen in 37.5% and 12.5%, respectively, though failure was seen in 50% of patients. Comparison of surgical outcome results between children and adults undergoing allografts was not done, due to the small number of patients.

In terms of time interval between injury to limbal stem cells and performing CLET, anatomical as well as visual outcomes were compared. No statistically significant difference was noted in patients operated in ≤6 months or >6 months (*p* values 0.77 and 0.81, resp.).

Depending upon the extent of limbal stem cell deficiency either partial or total, results of anatomical and visual outcomes had been evaluated. The difference was not statistically significant between the two (*p* value of anatomical and visual outcome between partial and total LSCD was 0.45 and 0.09, resp.).

In this study out of 62 operated patients prior surgery like symblepharon release and/or amniotic membrane transplant had been done in 24 patients. Out of these 24 patients, 20 patients were children in whom one surgery (in 14 patients) or two prior surgeries (in 3 patients) were done and among adult patients prior single surgery was documented in 7 patients. Amongst 62 operated patients complications were noted in 5 (8%) patients and all these patients were children. Complications encountered were corneal melting, recurrent symblepharon, and failed lamellar keratoplasty in one patient each and vascularized granulation tissue was noted in two patients. Second surgery was required in 3 (5.5%) patients. The site from where graft was taken for autograft had not shown iatrogenic stem cell deficiency in any of the patients throughout the follow-up period.

Due to an inadequate number of allograft patients comparative analysis of results between allograft and autograft could not be done.

## 4. Discussion

Ocular surface reconstruction in LSCD is one of the challenges in ophthalmic care. Treatment decision depends on the severity and laterality of deficiency as this indicates whether residual limbal stem cells are still present in the affected eye or an alternate source of limbal stem cells in the form of auto/allograft is required to be considered.

In our study the most common cause of limbal stem cell deficiency was thermochemical injury (96.3%) especially in children. This indicates that awareness about the thermochemical injuries and their dangerous impact on ocular surface should be increased among people.

Conjunctival limbal autograft was an accepted treatment modality in unilateral LSCD and was the only option available in the past [9]. However nowadays CLET is preferred over conjunctival limbal autograft in terms of less fellow eye complications like iatrogenic LSCD and filamentary keratitis and additionally it is an option for bilateral LSCD as well.

In the present study out of total of 62 patients, 87% of LSCD patients underwent autograft transplantation and 12.9% underwent allograft transplantation. Allograft had been avoided as far as possible due to the need of long-term systemic immune suppression, which has been documented to be associated with systemic side effect, affordability issues, and more chances of rejection and due to poor results in the previous literature [10].

In the present study, analysis of anatomical as well as visual success in paediatric patients has been documented in 66.6% and 21.2%, respectively, whereas in adults values were 80.9% and 38%, respectively. In the previous literature anatomical outcomes of 68–80% have been documented in adults [11].

Difference in outcome success in paediatric and adult age group is most probably attributed to increased magnitude of inflammation in children in response to any type of insult [12]. There was paucity of published literature on outcomes of limbal transplantation in paediatric age group. As far as we know only one study done by Sejpal et al. evaluated the outcomes of CLET in paediatric age group in which they had documented anatomical and visual outcomes of 46.7% and 54.2%, respectively. In Sangwan et al.'s study topical steroids had been given for 6 weeks in paediatric patients [6], whereas in the present study due to higher magnitude of inflammation expected in children topical steroids have been given for 8–12 weeks as compared to 6-week duration in adults. Noncompliance to medications, inadequate hygienic precautions, and difficulty to examine children properly without general anaesthesia may increase the chances of failure. In the present study although anatomical results are better than what has been reported earlier [7], visual success is lower. In paediatric age group, the reason of reduced visual success might be due to the amblyopic stimulus provided by sensory vision loss related to corneal involvement. In addition visual rehabilitation surgeries also get delayed in most of the cases due to persistent inflammation especially in paediatric age group.

Time duration between occurrence of limbal stem cells insult and surgery performed was evaluated in terms of anatomical and visual outcomes. Though poorer results were seen in patients where duration was <6 months, it was not statistically significant. In previous studies better outcomes have been documented if transplantation was done after 4 months of insult. This has been explained by saying that, in patients with ocular insult that occurred in less than 6 months, inflammation must be there which can further enhance the failure rates [13].

In the present study results have not shown any statistically significant difference in terms of overall anatomical and visual outcomes between children and adults. However complications rate was higher in paediatric age group and all the 4 patients where complete anatomical failure was noted were from paediatric age group. This highlights the importance of more frequent as well as thorough follow-up along with the use of topical steroids for a longer duration to get better results in children. Visual rehabilitation surgeries should be performed as early as possible once ocular surface gets stabilized to avoid amblyopia and poorer visual outcomes.

Further prospective as well as randomized controlled trials with larger sample size are required in this direction to confirm the present study results and also to compare various surgical modalities in children as well as adults with LSCD.

## 5. Conclusion

Cultivated limbal epithelial transplantation gives good long-term results in patients with LSCD and the outcomes are comparable in children and adults.

## Figures and Tables

**Table 1:** Various etiological factors for LSCD in children and adults.

Etiology	Age < 15 years *n* (%)	Age ≥ 15 years *n* (%)	Total *n* (%)
Thermochemical injury	33 (100)	19 (90.5)	52 (96.3)
Stevens-Johnson syndrome	0 (0)	1 (4.8)	1 (1.85)
Operated OSSN	0 (0)	1 (4.8)	1 (1.85)
Total	33	21	54

**Table 2:** Primary and secondary outcomes in terms of anatomical and visual success in children and adults.

Results	Age < 15 years			Age ≥ 15 years			*P* value
	*n* (%)			*n* (%)			
	Total success	Partial success	Failure	Total success	Partial success	Failure	
Anatomical success	22 (66.6)	7 (21.2)	4 (12.1)	17 (80.9)	4 (19.0)	0 (0)	0.23
Visual success	7 (21.2)	19 (57.5)	7 (21.2)	8 (38.0)	8 (38.0)	4 (19.0)	0.31
Total number of patients	33			21			
